# Hooked on Non-union: A Rare Case of Concurrent Scaphoid and Hook of Hamate Non-union

**DOI:** 10.7759/cureus.106634

**Published:** 2026-04-08

**Authors:** Aasiya Mannan, Jonathan Kent

**Affiliations:** 1 Orthopaedic Surgery, Royal Victoria Infirmary, Newcastle upon Tyne, GBR; 2 Orthopaedic Surgery, Cumberland Infirmary, Carlisle, GBR

**Keywords:** dual carpal fracture, hook of hamate fracture, non-union, scaphoid fracture, wrist injury

## Abstract

Carpal fractures are common orthopaedic injuries, with the scaphoid being the most frequently affected bone. Hamate fractures are uncommon and easily missed due to their subtle clinical presentation and difficulty in detection on standard radiographs, often being misdiagnosed as soft tissue trauma. Simultaneous involvement of the scaphoid and hamate is uncommon, and progression to dual non-union is even more so. We report a case of concurrent scaphoid and hook of hamate non-union to highlight the importance of early recognition and appropriate imaging to prevent delayed diagnosis and long-term complications.

A 31-year-old male presented with a painful wrist and tender anatomical snuffbox following a fall. Radiographs confirmed a scaphoid fracture, treated nonoperatively in a cast. Symptoms persisted after eight weeks; thus, a CT was performed, confirming a suspected scaphoid non-union and a hook of hamate non-union fracture. Management involved fixation of the scaphoid fracture and excision of the hook of hamate, with the patient pain-free and at baseline mobility at four months.

## Introduction

Hamate fractures are uncommon injuries, accounting for approximately 2-4% of all carpal fractures; however, this incidence is likely underestimated, as these injuries are frequently missed on initial radiographic assessment [1]. According to Milch’s classification, hamate fractures are divided into fractures of the hook (type I) and fractures of the body (type II), with hook fractures being more common. These injuries may occur in isolation or in the setting of high-energy trauma, where associated carpal fractures or disruptions are more likely [2].

Concurrent fractures of the scaphoid and hamate are rarely reported, and progression of both injuries to non-union is uncommon. To the best of our knowledge, no previous cases of simultaneous non-union of the scaphoid and hook of hamate have been reported in the literature. We present a rare case of dual carpal non-union and discuss the diagnostic challenges, management, and clinical implications.

## Case presentation

A 31-year-old male, a smoker, otherwise fit and well, presented to the emergency department with left wrist pain following a fall onto an outstretched hand earlier the same day. Clinical examination demonstrated swelling and marked tenderness over the anatomical snuffbox, painful restriction of wrist motion, and a positive axial thumb loading test. Neurovascular examination was normal.

**Figure 1:**
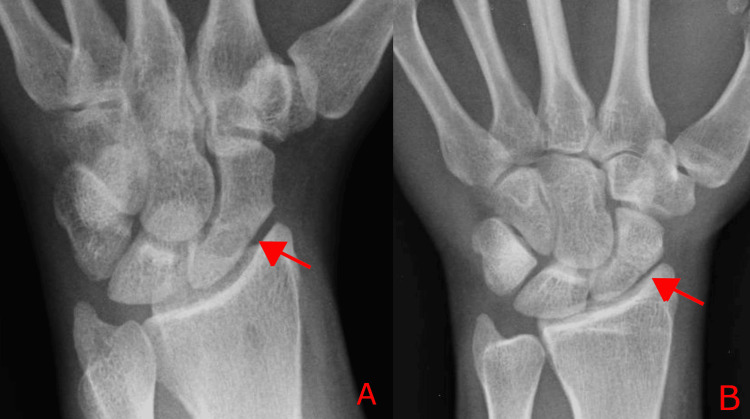
Initial wrist radiographs demonstrating a minimally displaced scaphoid fracture (A–B) Posteroanterior in ulnar deviation and posteroanterior radiographs. Arrows indicate the scaphoid fracture.

Initial plain radiographs demonstrated a minimally displaced scaphoid fracture, with no other obvious carpal injuries (Figure 1). The patient was referred to the upper limb clinic and managed nonoperatively with immobilisation in a scaphoid cast.

**Figure 2:**
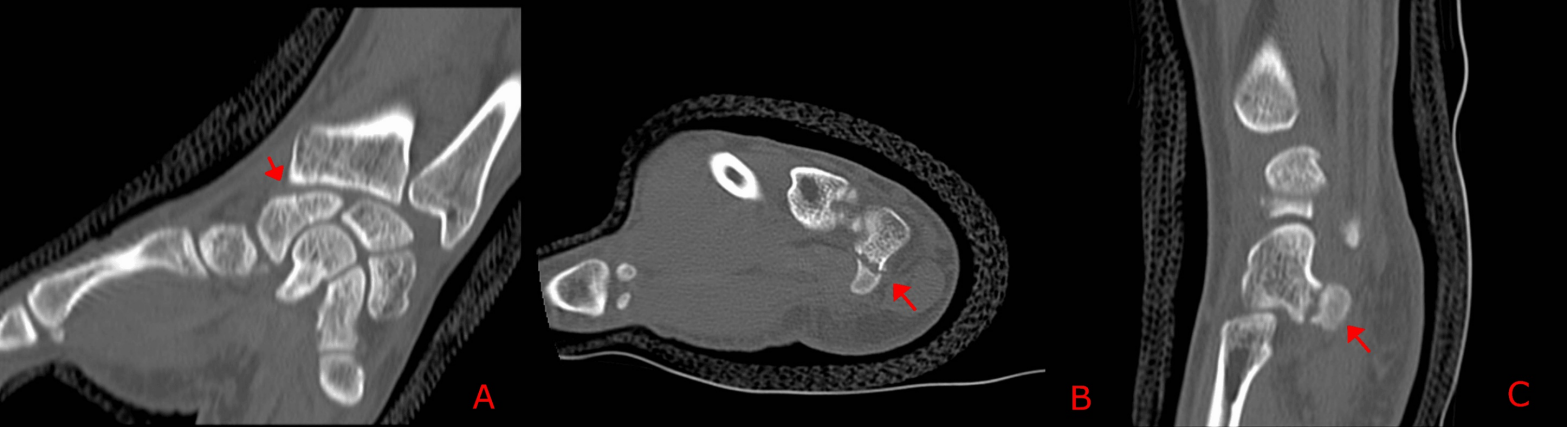
CT of the wrist at eight weeks demonstrating non-union fractures of the hook of hamate and scaphoid (A) Coronal CT images demonstrating a non-union scaphoid fracture (arrow). (B) Axial and (C) Sagittal CT images showing a non-union fracture of the hook of hamate (arrows). CT: computed tomography

At the eight-week review, the patient remained symptomatic. Repeat radiographs showed insufficient evidence of healing. On examination, there was persistent snuffbox tenderness with axial thumb loading, as well as new tenderness over the hypothenar eminence with point tenderness localised to the hook of hamate. The scaphoid cast was reapplied, and a CT scan was performed one week later, confirming a scaphoid non-union (Figure 2C-2D) and demonstrating a non-union fracture of the hook of hamate (Figure 2A-2B).

**Figure 3:**
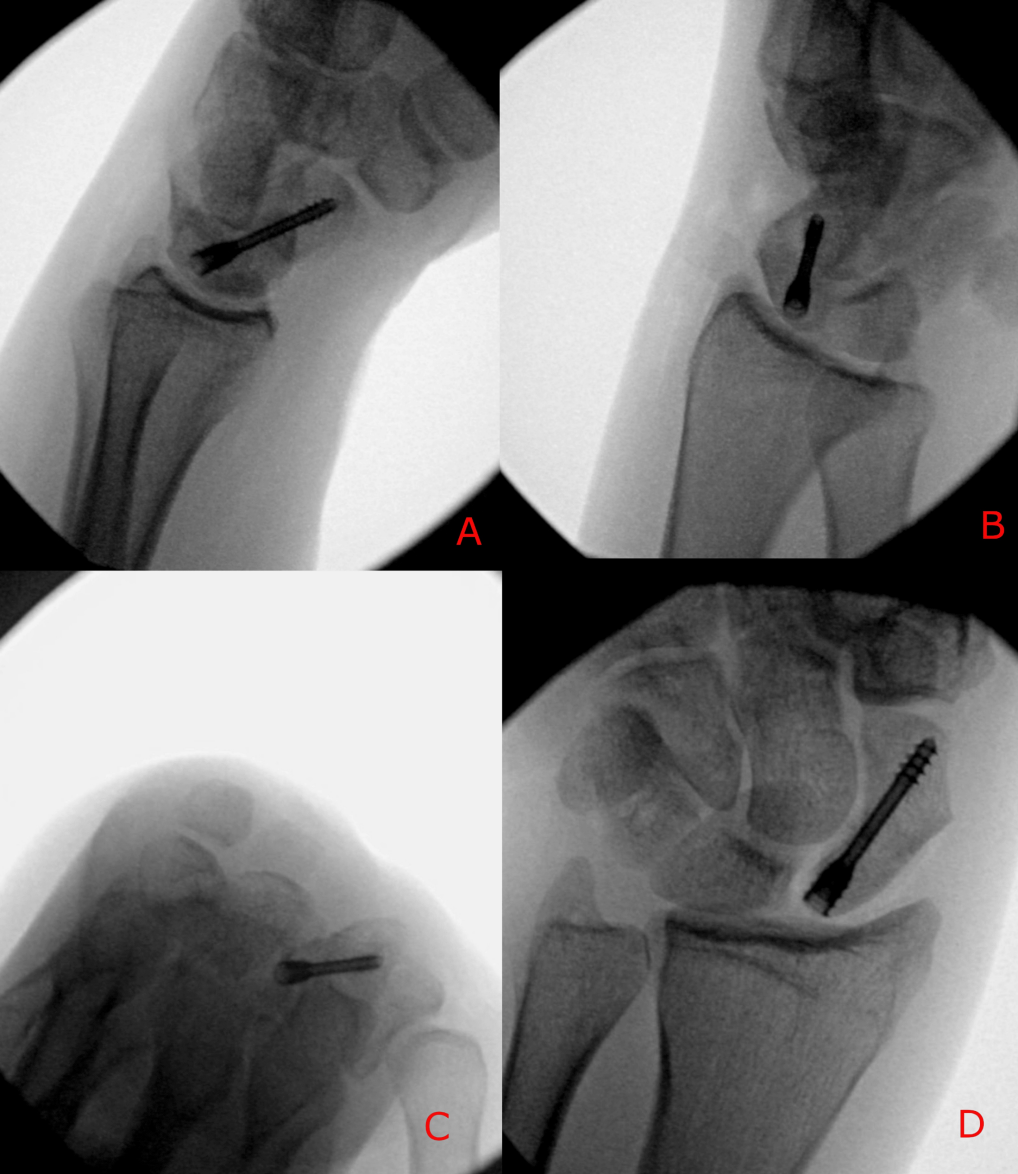
Intraoperative fluoroscopic images demonstrating scaphoid fracture fixation (A–D) Lateral, oblique, carpal tunnel views and posteroanterior radiographs showing placement of a headless compression screw across the scaphoid fracture.

The patient subsequently underwent surgical management consisting of percutaneous fixation of the scaphoid fracture with a headless compression screw and excision of the hook of hamate (Figure 3A-3D). Excision, as opposed to fixation of the hook of hamate, is a more reliable option with an easier technique, more predictable results, and fewer complications.

Surgical technique

A dorsal approach to the wrist was used, with a 2 cm incision in line with Lister’s tubercle. The extensor retinaculum was opened between the third and fourth compartments, and the wrist capsule was incised. The scapholunate ligament was identified. Under fluoroscopic guidance, a guidewire was inserted into the scaphoid along its axis, adjacent to the ligament. A cannulated drill was then used over the guidewire to prepare for screw fixation. An appropriately sized headless compression screw was inserted and fully buried under direct vision. The wound was closed in layers. Bone grafting was not performed due to the short duration of non-union and the absence of deformity.

A separate incision was made over the hypothenar eminence. The pisohamate ligament was released. The ulnar nerve and artery were identified and carefully followed distally to the hook of hamate. While preserving neurovascular structures, the hook of hamate was excised and smoothened using a nibbler. The wound was closed after haemostasis was achieved.

Postoperative course

**Figure 4:**
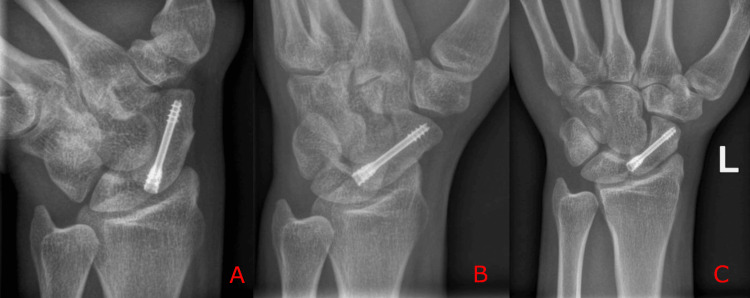
Postoperative six-month wrist radiographs demonstrating healed scaphoid fracture (A–C) PA in ulnar deviation, oblique and posteroanterior radiographs showing complete fracture healing with the headless compression screw in situ.

At eight weeks postoperatively, the patient was almost pain-free, and radiographs demonstrated progression toward scaphoid union. He was referred for physiotherapy and, at the six-month follow-up, reported a satisfactory outcome, with resolution of pain and return to baseline wrist mobility (Figure 4A-4B).

## Discussion

Hamate fractures are rare injuries and are easily missed, particularly when a more clinically apparent injury, such as a scaphoid fracture, is present. The hook of hamate is a curved projection arising from the hamate body and protrudes into the hypothenar region. Fractures of the hook are commonly associated with sports involving a strong grip, where shearing forces from the flexor tendons of the ring and little fingers are transmitted to the hook, or following a fall onto an outstretched hand [3].

Scaphoid fractures typically present with pain and tenderness in the anatomical snuffbox and are at risk of non-union due to their retrograde blood supply. Although concomitant scaphoid and hook of hamate fractures are rare, their coexistence may be under-recognised due to limitations of plain radiography and overlapping clinical features. Progression of both fractures to symptomatic non-union represents an exceptionally uncommon sequence of events.

Patients with hook of hamate fractures often report hypothenar pain aggravated by grasping, point tenderness on deep palpation, hypothenar eminence tenderness, and pain with dorso-ulnar deviation of the wrist. Ulnar nerve paraesthesia, flexor tendinitis, or carpal tunnel-type symptoms may also occur. Delayed presentation may result in complications such as flexor tendon tenosynovitis or rupture [4]. On examination, the patient may have a positive Tinel's test over Guyon's canal, or you may perform a hook of hamate pull test (holding the hand in ulnar deviation as the patient flexes the DIP joints of the ulnar against resistance, thus eliciting pain).

Diagnosis of hook of hamate fractures using standard radiographs is challenging, even when clinical suspicion is high. Specialised views, including carpal tunnel and oblique projections, may improve detection. CT is considered the most reliable modality for diagnosing hook of hamate fractures. At the same time, magnetic resonance imaging can demonstrate associated soft-tissue injury and vascular compromise, and help differentiate acute fractures from congenital variants such as hamulus proprius [5-6].

A retrospective imaging study demonstrated that 10.3% of patients with scaphoid fractures had a concomitant hook of hamate fracture identified on CT or MRI, many of which were minimally displaced and not visible on initial radiographs [7]. This finding highlights the importance of careful clinical examination and consideration of advanced imaging when managing scaphoid fractures with persistent or atypical symptoms.

Management of scaphoid fractures may be nonoperative or operative depending on displacement, patient factors, and healing response. Hook of hamate fractures can be treated conservatively with immobilisation or surgically with open reduction and internal fixation or fragment excision. If the patient is presenting with neurological symptoms, you may also consider a decompression of Guyon's canal alongside excision.

Due to the high reported rates of non-union with conservative management, several authors advocate early surgical intervention, particularly excision of the hook, which has been shown to reliably relieve symptoms [8].

Previous reports of concomitant scaphoid and hamate fractures are exceedingly rare. Komura et al. described a simultaneous fracture of the scaphoid waist and the hook of hamate that was identified acutely and treated surgically, without progression to non-union [9]. Other published cases have reported combined scaphoid fractures with fractures of the hamate body rather than the hook. Jones and Hems reported a simultaneous fracture of the hamate body and distal pole of the scaphoid following trauma. At the same time, Yalcinkaya et al. described a rare wrist injury involving fractures of the hamate body and scaphoid waist [10-11]. Notably, in all previously reported cases, the fractures were identified in the acute setting and managed before progression to non-union.

To the best of our knowledge, this is the first reported case of concurrent non-union of both the scaphoid and the hook of hamate, highlighting the consequences of delayed or missed diagnosis and reinforcing the need for vigilance when assessing wrist injuries.

## Conclusions

Concurrent non-union of the scaphoid and hook of hamate is an uncommon condition and may result from delayed or missed diagnosis. This report highlights the need to maintain a high index of suspicion for multiple carpal fractures in patients and that careful clinical examination is always required. When suspected, an expert opinion is important to prevent long-term functional impairment. Additionally, early use of appropriate imaging (CT/MRI) should be requested when there is enough clinical evidence and non-conclusive radiographs.
